# The Emerging Roles of AI in Self-Directed Stress Management: Systematic Review

**DOI:** 10.2196/90709

**Published:** 2026-06-24

**Authors:** Mary Kamillah Grace Reyes, Shauna Sha Min Teo, Andree Hartanto

**Affiliations:** 1 School of Social Sciences, Singapore Management University, Level 4, 10 Canning Rise, Singapore, 179873, Singapore, 65 68281901, 65 68280423

**Keywords:** artificial intelligence, chatbots, conversational agents, digital mental health, stress management, self-directed stress management, self-guided intervention, psychoeducation, stress monitoring, Preferred Reporting Items for Systematic Reviews and Meta-Analyses, PRISMA, systematic review

## Abstract

**Background:**

Stress is widespread and carries substantial mental health, social, and economic burdens. Yet, access to clinician-led stress management remains constrained by service capacity, cost, and stigma. In response, artificial intelligence (AI)–enabled tools have rapidly proliferated as scalable, self-directed options. However, evidence on how these systems support stress management outside formal clinical settings remains fragmented.

**Objective:**

This systematic review aimed to synthesize empirical evidence on how AI-enabled technologies are used for self-directed stress management. We mapped the emerging functions of these tools, the psychological frameworks informing their design, the populations and settings studied, and the outcomes reported.

**Methods:**

We conducted a PRISMA (Preferred Reporting Items for Systematic Reviews and Meta-Analyses)–compliant systematic review of English-language studies published between 2000 and 2025. Six databases were searched (APA PsycINFO, PubMed, MEDLINE, Scopus, Web of Science Core Collection, ProQuest, and Google Scholar).

**Results:**

Of 3008 records identified, 35 studies met the inclusion criteria. The methodological quality of included studies was critically appraised using the Mixed Methods Appraisal Tool (version 2018). Findings illustrated that AI-supported stress management can operate through 5 core functions, including psychological intervention, behavioral support, psychoeducation, companionship, and emotional support, and stress monitoring, detection, and triage. Across the reviewed studies, these functions supported self-directed stress management by helping users identify stress, regulate responses, and engage in coping outside formal clinical care.

**Conclusions:**

AI-enabled systems show preliminary promise for supporting self-directed stress management through multiple user-facing functions grounded in established psychological frameworks.

## Introduction

### The Emerging Roles of Artificial Intelligence in Self-Directed Stress Management

#### Overview

From minor hassles to major crises, everyday life is saturated with stress, commonly understood as a state of worry or mental tension caused by difficult situations [1]. Amidst the COVID-19 pandemic, stress symptomatology was estimated to affect up to 25% of individuals [2,3]. Global estimates indicate that stress prevalence ranges from approximately 13% to nearly 40% across population-level surveys and meta-analytic studies, with approximately 1 in 3 adults reporting daily stress and most countries showing worsening trajectories over the past decade [4-7]. These figures are often magnified in at-risk groups, such as informal caregivers. Meta-analytic evidence indicates that informal caregivers face a substantial mental health burden, with many reported to meet diagnostic criteria for mental health conditions, and nearly half experience clinically significant caregiver burden [8,9]. Therefore, stress can act as a symptom of existing difficulties and as a risk factor that worsens long-term mental health trajectories. However, scalable and flexible support that can accommodate diverse needs remains limited by both system-level and individual-level barriers. For instance, constrained service capacity means that groups such as students experience high stress but low uptake of treatment [10,11]. Meanwhile, financial, logistical, and stigma-related obstacles disproportionately impede access to clinical and counseling care, contributing to poorer mental health outcomes [12-14].

In light of this, self-directed stress management has gained traction as a practical alternative. Such approaches parallel self-help interventions, defined as standardized, evidence-based programs that participants implement independently of professional therapists or guides [10,15]. These self-directed solutions can enhance accessibility while offering greater autonomy and convenience, especially for those facing structural or social barriers to conventional care. Digital mental health interventions targeting youth have shown potential to achieve sustainable improvements in stress and anxiety outcomes by providing personalized, accessible, and culturally responsive resources. However, high dropout rates, limited long-term data, and the inherently low accountability of self-directed digital interventions [16,17] identify a clear need for novel strategies for supporting continued use and intervention efficacy.

#### AI and Self-Directed Stress Management Interventions

Given these limitations, artificial intelligence (AI) offers capabilities to address gaps in current self-directed stress management programs. Large language models (LLMs), natural language processing (NLP), and related machine learning approaches have become increasingly ubiquitous in many sectors. In this context, a key strength of these intelligent models is their ability to interpret complex user data and translate it into tailored, context-specific responses [18-20]. Leveraging these capabilities could enable more accessible and efficient delivery of stress management solutions that are responsive to individual needs. In broader health care contexts, AI has been proposed and increasingly applied to support personalized monitoring, reduce selected workflow burdens, and assist routine tasks [21-23]. These precedents suggest that AI-enabled models can harness similar efficiencies for independent stress self-management.

At the intervention level, workplace internet-based stress management interventions have demonstrated substantial reductions in perceived stress [24] as well as cost-effective benefits [24,25]. AI-based systems further enhance these interventions by offering greater scalability, consistent availability, and anonymity [26-28], which may lower barriers for individuals who are hesitant to engage in face-to-face stress management programs. Conversational agents exemplify this potential by enabling dialogue that users experience as more therapeutic, personalized, and autonomous [17,29,30]. Some evidence suggests that users may be more willing to disclose sensitive information to nonhuman agents, partly because of reduced perceived judgment [17,31-33]. In addition, intelligent conversational models do not require ongoing clinician involvement, allowing them to scale far more efficiently than human-delivered or clinician-supported digital interventions [34-36]. Collectively, these capabilities indicate that AI-driven self-directed stress management tools can extend beyond static guidance to provide adaptive, ongoing support that responds to technological advances and broader societal needs for accessible, flexible, and scalable approaches to stress management.

#### Applications of AI for Self-Directed Stress Management

One prominent application of AI in this context is the use of physiological and behavioral signals to monitor stress. Specifically, stress monitoring is based on measuring physiological changes and comparing them with baseline metrics [37-39]. The symptomatology of stress involves consistent and predictable physiological changes, such as heart rate variability; AI may be used to detect and model these physiological signs to infer when an individual is under stress [40]. Machine learning models trained on such health data can classify stress states with promising accuracy [41] and, when embedded in wearable or smartphone-based sensors, could provide continuous, unobtrusive monitoring across daily contexts [38,39]. This addresses a core limitation of traditional self-report measures, which capture only brief, time-bound snapshots of experience [39,42]. Related literature on digital phenotyping shows that passive smartphone-sensor data can be used to infer behavioral and symptom patterns, enabling the matching of individuals to mental health applications that suit specific needs [43,44], offering a route to more personalized and timely self-help support.

Beyond monitoring physiological symptoms, AI can also support behavior change processes that reduce stress in the long term. Intelligent systems are increasingly used to deliver habit tracking tools, adaptive nudges, and tailored coping suggestions, drawing on broader developments in digital behavior change technologies [45-47]. In this context, “user-centered” and “person-based” design approaches have become central to sustaining behavioral patterns in similar interventions [48]. In practice, virtual therapists and chatbots may offer relatively discreet spaces, reducing perceived stigma and the sense of judgment that often deters help-seeking [35,49]. Empathy-oriented conversational agents take this further by analyzing user sentiment and generating emotionally attuned responses [50]. Such intelligent dialogue platforms have shown promise in reducing student stress through supportive, user-centric conversation and increased perceived trust in the conversational agent [50-52].

At the same time, the broader adoption of AI in mental health raises substantial concerns, as generative and predictive models are inherently nondeterministic and may produce unreliable or erroneous outputs. Such risks are compounded when these probabilistic models are trained on noisy, incomplete, or low-fidelity data from consumer-grade devices unable to match the reliability of clinical-grade systems [38,53]. Data privacy concerns are especially salient for multimodal AI models that rely on facial images, voice recordings, or other sensitive user data [54,55]. Under regulations such as the Health Insurance Portability and Accountability Act (HIPAA), patient health information held by covered entities is strongly protected; however, most existing regulatory and data protection frameworks have yet to fully consider how AI developers and vendors collect, use, and share mental health–related data [55-57]. In practice, many of these systems are trained on datasets that are limited in scale and diversity, with systematic underrepresentation of minority groups [58-60]. This pattern of limitations systematically biases model predictions and performance for users in marginalized populations, potentially exacerbating existing mental health care inequalities [23,35,58-60]. As a result, appraising the value and impact of AI-driven interventions for stress management requires rigorous scrutiny of how these systems are conceptualized, designed, trained, and evaluated for different population needs.

### This Research

Despite rapid growth in AI-enabled mental health tools, evidence on AI for self-directed stress management remains fragmented. Existing studies vary widely in the types of AI used, the theoretical frameworks guiding development, and the stress-related functions targeted (eg, monitoring, psychoeducation, and behavior change), and there has been no systematic synthesis focused on autonomous use outside formal clinical settings. This review addressed this gap through a systematic examination of empirical studies published between 2000 and 2025 that used AI-integrated platforms independently by individuals to cope with stress outside formal clinical care. Specifically, we identified the main categories of AI tools evaluated to date and noted the populations and settings they target. Finally, we analyzed the functions by which these tools support self-directed stress coping. In doing so, we aimed to provide an integrated overview of existing approaches and lay a foundation for future work on responsible, user-centered AI in self-directed stress management.

## Methods

### Transparency and Openness

We conducted this systematic review in accordance with the PRISMA (Preferred Reporting Items for Systematic Reviews and Meta-Analyses) guidelines (Checklist 1) [61]. The review protocol, including the search strategy and planned synthesis framework, was submitted for registration on PROSPERO (registration ID: CRD420251135780). Furthermore, citation management, record deduplication, and metadata extraction were conducted using Zotero version 7.0.24 (Corporation for Digital Scholarship) [62]. Relevant files, namely screening records and extraction data used in this work, will be made publicly available upon publication.

### Search Strategy

A systematic search strategy was jointly developed by the first 2 authors (MKGR and SSMT) and conducted across 6 electronic databases: APA PsycINFO, PubMed or MEDLINE, Scopus, Web of Science Core Collection, ProQuest, and Google Scholar. The long-string series of keywords, (“artificial intelligence” OR “A.I.” OR AI OR GPT OR chatbot OR “large language model” OR LLM*) AND (stress OR “stress management” OR “stress response*” OR (“self-directed” AND stress*) OR “de-stress*” OR “self-care”), was applied to APA PsycINFO, PubMed, Scopus, and Web of Science. All records were extracted from these 4 databases. For supplementary searching in ProQuest and Google Scholar, a shortened query with the same Boolean structure was used, namely (“artificial intelligence” OR AI OR GPT OR LLM OR chatbot) AND (stress OR “stress management” OR “stress response*” OR “de-stress*"). Only the first 100 results were retrieved based on relevance. Records were limited to studies published in English from 2000 to 2025 that involved human participants, including early online publications where available. Exclusion filters were applied to exclude medical, biological, and clinical trial domains unrelated to self-directed stress management. The search and extraction of records were conducted on September 4, 2025.

### Screening Criteria and Calibration

This review used a 2-stage screening process consisting of (1) title and abstract screening and (2) full-text screening. Both the first and second authors independently reviewed studies from all 6 sources, with a focus on human subjects who independently use AI or AI-enabled agents for personal stress management. We excluded records that involved AI and stress management in purely clinical settings, AI use in non–stress-related settings, and non–human-related stress (eg, stress on crop rotation). Records were retained according to the following inclusion criteria:

1. The record was written in English.
2. The record was unique and not a duplicate of any prior entry.
3. The record was not retracted or withdrawn at the time of screening.
4. The record explicitly addressed self-directed or personal stress management, rather than clinician-led, medically supervised, or biomarker-based clinical interventions.
5. The record provided sufficient information to obtain full-text access.

A trial set of 100 records was independently screened to calibrate inclusion decisions and ensure consistency. Following this calibration, the remaining records were screened independently. Records with insufficient abstract detail but retrievable full text were advanced, whereas records with no abstract and insufficient retrievable information were excluded. Records meeting inclusion criteria at the title and abstract level proceeded to full-text review. Similarly, at this subsequent full-text screening stage, a trial set of 50 records was undertaken to ensure alignment, after which the same inclusion and exclusion criteria as those used in title and abstract screening were applied, with the addition of the following criteria:

1. The record was peer reviewed and published.
2. The record did not pertain to interventions delivered in conventional clinical mental health settings.
3. The record explicitly involved AI use or AI-assisted technology (eg, chatbots, LLMs, and AI-enabled apps) for stress self-management.
4. The record noted that AI use was “self-directed,” ie, initiated and managed by the user without clinician or therapist oversight.
5. The record presented empirical data (quantitative, qualitative, or mixed methods) and collected data from human participants.

### Data Extraction and Synthesis

Data extraction was conducted independently by both the first and second authors, with discrepancies resolved through discussion. As the included records comprised both qualitative and quantitative studies, thematic analysis was used to identify and categorize recurring themes in how AI supports stress management. This approach was appropriate because it enabled findings from qualitative, quantitative, and mixed method studies to be integrated into a coherent whole, consistent with guidance for mixed methods systematic review [63]. An exploratory coding procedure was applied to identify recurring patterns, emerging categories, and overlapping conceptual discussions within the agreed extraction criteria. A trial set of 20 records was screened to ensure consistency of data for extraction. When consolidating outcome data across heterogeneous study designs, we prioritized textual descriptions of reported effects; where quantitative results were available, these were also mapped onto a common "direction of effect" rubric (improvement, no or minimal change, and worsening), following recommendations for thematic synthesis in systematic reviews of mixed evidence [64,65].

To synthesize data from both qualitative and quantitative records, we followed Thomas and Harden’s [66] procedure for thematic synthesis. After confirming the final set of included records, both first and second authors reviewed all records again to develop codes for the text and descriptive themes. We initially analyzed each record against our full-text inclusion criteria, focusing on the forms of AI used to manage stress, the notable frameworks on which AI-enabled agents were trained to provide help, and the significant or nonsignificant results of interventions. The first and second authors then discussed our preliminary findings to produce a list of themes that address the focus of our research: how AI models support stress management. Themes were internally refined to ensure coherence and clear differentiation. Both the first and second authors independently reviewed the final included records and categorized them. In addition, following the data extraction protocols of Aromataris et al [67] for umbrella review, the following were also extracted from each record: (1) author and year of publication, (2) journal or publication, (3) country (of sample), (4) study type, (5) study pool, (6) sample size, (7) mean age (years), (8) age range (years), (9) intervention name and description, (10) intervention framework, and (11) key findings.

## Results

### Overview of Search and Inclusion Results

**Figure 1.**
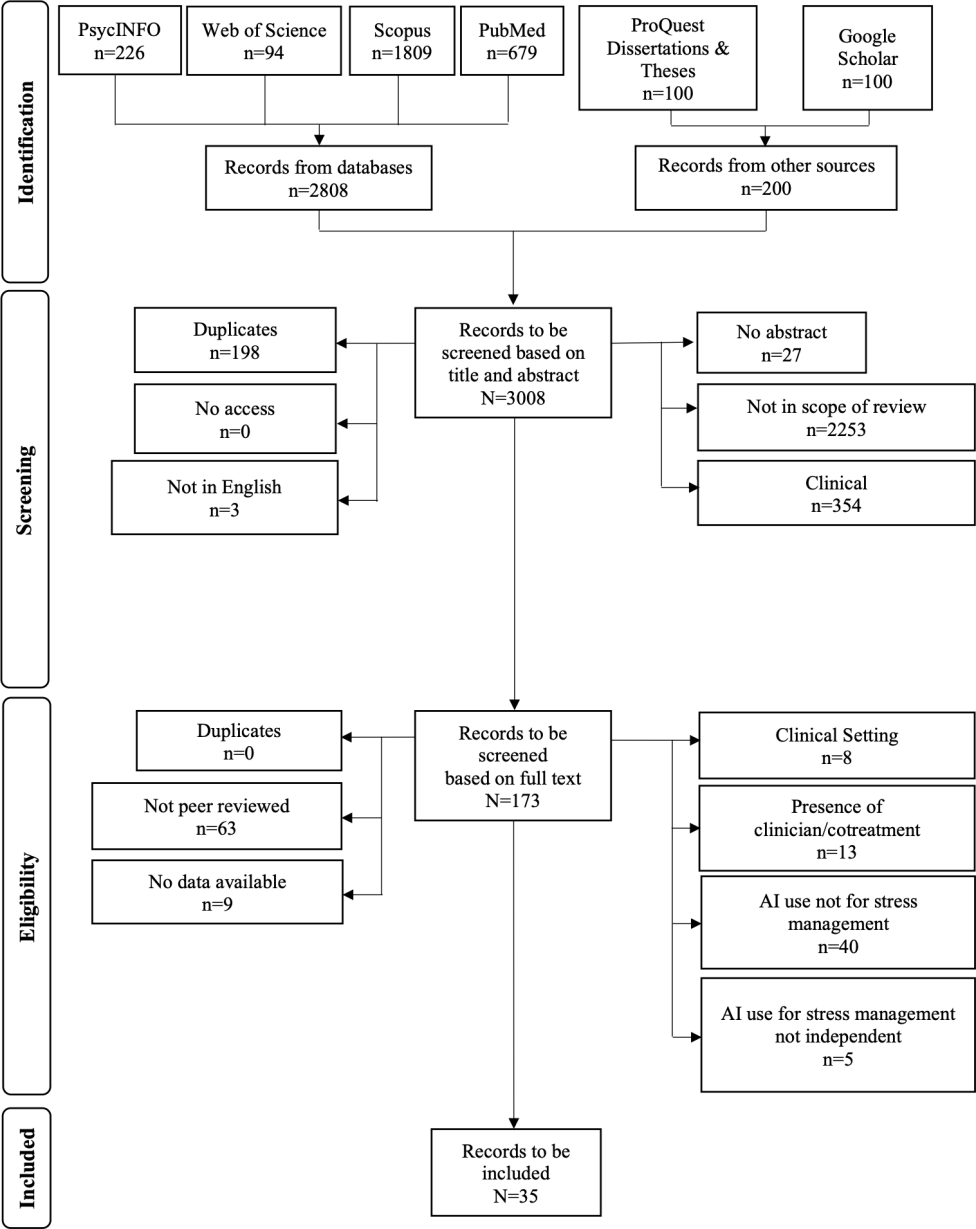
PRISMA (Preferred Reporting Items for Systematic Reviews and Meta-Analyses) diagram outlining the systematic search process. AI: artificial intelligence.

The search string yielded 3008 records (Figure 1). The full list of all unique records identified and screened (N=2810), including those advanced to full-text review (n=173) and excluded full-text articles with reasons (n=138), is available in Multimedia Appendix 1. After removing duplicates (198/3008, 6.58%), 2810 (93.42%) unique records remained. This review proceeded in 2 phases. In the first phase, both authors reviewed the titles and abstracts of all 2810 records to determine eligibility for the full-text phase based on the criteria outlined in the “Methods” section. A total of 2637 (93.84%) records were excluded after title and abstract screening, retaining 173 (6.16%) records for full-text screening. Specifically, 27 (1.02%) records lacked an abstract, 354 (13.45%) were clinical studies not explicitly related to stress self-management, 3 (0.11%) were not in English, and 2253 (85.44%) were outside the scope of this review. In the second phase, the 173 included records were reviewed in full by both authors. During this full-text screening phase, 63 (36.42%) records were removed for not being peer reviewed or in preprint, 8 (4.62%) records were studies set in purely clinical settings (ie, set in conventional therapeutic settings), 13 (7.51%) records involved the presence of a clinician or AI use for stress relief that was co-administered with clinical treatment, 40 (23.12%) records had AI use not for stress management, and 5 (2.89%) records did not have independent use of AI tools for stress management. During the data extraction and thematic analysis, a further 9 (5.20%) records were removed due to insufficient data. A total of 35 (20.23%) records were included in the final review. The overall agreement rates for title and abstract screening (2426/2810, 86.30%) and for full-text screening (138/173, 79.77%) were adequate, with data extraction agreement at >70%. Disagreements were resolved at the end of each stage through discussion with both authors.

### Overview of Included Studies

This review synthesized 35 studies, including 3 (8.57%) qualitative studies, 11 (31.43%) randomized controlled trials, 6 (17.14%) nonrandomized quantitative studies, 4 (11.43%) quantitative descriptive studies, and 11 (31.43%) mixed methods studies. These included studies examined AI-enabled tools for self-directed stress management across chatbots, mobile apps, embodied agents, and other digital platforms. Table 1 summarizes the key characteristics of the included records, including study-level information, intervention frameworks, AI architectures used, and sample characteristics where reported. Sample characteristics include the study pool or demographic focus of each sample (eg, employed nurses, fathers, and university students), as well as sample size, mean age, and age range. AI architecture classifications for each study (eg, LLM-based, rule-based, and hybrid) are also included in Table 1.

**Table 1.** Characteristics of included records.

Author, year of publication	Journal or publication	Country/Region	Study type	Study pool	Sample size	Mean age (y)	Age range (y)	Intervention framework	AI^a^ architecture used	Key findings
Alanazi et al, 2025 [68]	*Scientific Reports*	Saudi Arabia	Mixed methods	Children with Down syndrome and caregivers of children with Down syndrome	N=130 (n=7 and n=123)	—^b^	3‐18 (children) and ≥18 (caregivers)	Mixed (CBT^c^-, occupational-, and behavior-oriented components)	Several multimodal AI systems, combining computer vision, NLP^d^, and machine learning	Moderate-large improvements were observed in children’s mobility (*d*≈0.65), communication (*d*≈0.72), and daily living or household tasks (*d*≈0.83); carers reported reduced caregiving stress and greater child independence, engagement, and confidence, although adoption was limited by cost, lack of support, and technical issues, with qualitative reports also indicating subjective stress reduction.
Alanezi, 2024 [69]	*Journal of Multidisciplinary Healthcare*	Saudi Arabia	Qualitative	Mixed adult population	n=24	—	≥18	CBT-based psychoeducation	ChatGPT-3.5 or / LLM^e^-based conversational AI	Participants reported using AI mental health tools primarily for psychoeducation (~60%), emotional support, and feeling heard (~50%), goal setting or motivation, and accessing mental health resources; perceived benefits included CBT-like reframing, coping guidance, and self-monitoring, while concerns centered on limited assessment accuracy, data privacy or ethics, and cultural-linguistic mismatch.
Allan, 2024 [70]	*Behaviour and Information Technology*	New Zealand	Quantitative nonrandomized	SME^f^ owners and managers	N=31 (n=14; n=17)	36.9	21‐57	Stress Mindset Theory [71]	Rule-based decision-tree chatbot with keyword matching	A brief conversational agent intervention produced very large gains in a “stress-is-enhancing” mindset (eg, SMM^g^ scores approximately doubled, *d*≈2.19) and large improvements in self-reported work performance (*d*≈1.59), with very high engagement (100% adherence and 86% wanting continued access) and participants attributing productivity gains to stress-reframing processes.
Baek and Cha, 2025 [72]	*Worldviews on Evidence-Based Nursing*	South Korea	Quantitative randomized controlled trials	Employed nurses	N=120 (n=40; n=40; n=40*)*	31.55; 31.88; 32.05	≥18	WHO^h^-based program integrating mindfulness, ACT^i^, storytelling, and laughter therapy	AI recommendation algorithm using similarity scoring and neural-network updating	Across 4 weeks, the AI-supported intervention (and especially self-selected use) produced significant reductions in client-related and personal burnout and stress responses, with job stress decreasing in all conditions; stress-response reductions were strongest in the AI and self-selection groups, but not evident in the minimal-control group.
Cho et al, 2024 [73]	*Journal of Medical Internet Research*	South Korea	Quantitative nonrandomized	Employed nurses	N=603 (n=293; n=10; n=300)	32.81	≥18	WHO-based mindfulness, ACT, storytelling, laughter therapy modules	Collaborative-filtering recommender with artificial neural network updates	Burnout, job stress, and stress responses significantly decreased from baseline through posttest assessments (*P*<.001), with multicomponent modules (mindfulness, ACT, storytelling, laughter therapy) each improving different burnout facets and stress indices; satisfaction increased as the AI optimization progressed, and reductions in job stress and stress response were already evident after 2 weeks.
Fuhrmann et al, 2025 [74]	*Scientific Reports*	Germany	Quantitative randomized controlled trials	Mixed adult population	N=166 (n=56; n=56; n=54*)*	25.39; 23.25; 23.76	18‐60	CBT-informed stress management (HRV^j^ biofeedback+CBT elements)	Sensor-based signal processing using accelerometer-derived HR^k^ biofeedback and facial emotion recognition	Both versions of the stress management app improved perceived stress, emotion regulation, and well-being, but the HR-based biofeedback version yielded significantly greater reductions in perceived stress than the waitlist control (significant time× condition interaction on PSS^l^), with high usability ratings.
Gabrielli et al, 2021 [75]	*JMIR mHealth and uHealth*	Italy	Mixed methods	University students	N=71	20.6	18‐34	CBT, mindfulness, positive psychology	Scripted rule-based chatbot with predefined response options	Use of the app was associated with significant decreases in perceived stress (PSS-10) and severe anxiety symptoms, alongside improvements in mindfulness facets (describing, nonjudging); stress levels shifted from high to low categories over time, with moderate engagement (≈78 interactions; 58% completion).
Hiller et al, 2025 [76]	*JMIR mHealth and uHealth*	Germany	Qualitative	Mixed adult population	N=61 (n=27; n=34)	30.5; 20.38	18‐59	Ecological momentary intervention or just-in-time adaptive intervention with CBT and ACT components	Predictive time-series machine learning for adaptive EMI^m^ delivery	A 4-week digital intervention significantly improved well-being and reduced perceived stress and depressive symptoms; qualitative feedback highlighted increased mental health awareness, the ability to identify stress triggers, and the value of ongoing structure, support, and emotional guidance.
Huang et al, 2022 [77]	*International Journal of Social Robotics*	Taiwan	Quantitative nonrandomized	Mixed adult population	N=122 (n=23; n=40; n=59)	25; 22.1; 23.87	—	Study-specific human-robot Interaction framework (ability, appropriateness, and validity)	Hybrid NLP system using knowledge graph, Bayesian network, neural network, and AIML^n^ chatbot	Participants rated the robot’s stressor inference accuracy highly (~4.4/5) and judged its emotional support responses as more appropriate than those of a baseline chatbot; they perceived the agent as understanding their feelings, providing valid and reliable emotional support, and making self-disclosure easier.
Indrayanti et al, 2025 [78]	*SSM–Mental Health*	Indonesia	Mixed methods	University students	N=32 (n=16; n=16)	20.21	19‐22	PFA^o^	GPT-3 chatbot with NLP-based sentiment detection	Compared with controls, the PsyBot condition significantly reduced loneliness and improved well-being over 2 weeks, indicating that the chatbot was more effective than controls in alleviating social isolation.
Jiang and Yang, 2025 [79]	*BMC Psychology*	China	Quantitative randomized controlled trials	Middle-school students	n=65	15.24; 15.75	14‐18	CBT (Elomia) with Mindfulness and PFA-derived components	Hybrid recommendation and chatbot system using NLP and machine learning	Relative to controls, the intervention produced significant and sustained reductions in somatic symptoms, anxiety and insomnia, social dysfunction, and severe depression, reflecting overall alleviation of psychological distress, while the control group showed no meaningful change.
Keung and So, 2025 [80]	*Frontiers in Psychology*	Hong Kong	Quantitative randomized controlled trials	University students	N=110 (n=55; n=55)	29.93; 28.13	18‐71; 18‐60	CBT-based supportive chatbot	GPT-4 or LLM-based counseling chatbot	Stress levels decreased from pre- to post-test in both AI and human support conditions, regardless of whether the chatbot’s identity was revealed, but higher “perceptual fear” of AI predicted lower perceived support quality; those with lower fear evaluated the chatbot more favorably.
Ko and Woo, 2025 [81]	*JMIR mHealth and uHealth*	South Korea	Quantitative descriptive	Population not specified	N=7 (raters)	–	–	Mixed (CBT, health information provision, and physiological monitoring)	Mixed app ecosystem with algorithmic chat, AI mood checking, and recommender features	Among commercial apps, those providing rich mental health information, counseling services, and multiprovider details achieved the highest uMARS^p^ scores (17/41 apps >4.0, range of 3.08‐4.08) and were preferred by users, who particularly valued 24-hour access and not having to rely on a single provider.
Lai et al, 2025 [82]	*Journal of Nursing Research*	Taiwan	Quantitative randomized controlled trials	Employed nurses	N=80	33.6	≥18	Stress appraisal and coping theory+CBT	Hybrid GPT-supported chatbot embedded in structured intervention content	Relative to controls, the intervention produced significant improvements in sleep disturbance, perceived stress, anxiety, and depressive symptoms by 6-week follow-up (all *P*<.001); perceived stress was already lower in the intervention group by 3 weeks, whereas the control group showed a slight increase in stress over time.
Lee and Hahn, 2024 [83]	*Frontiers in Psychology*	South Korea	Quantitative nonrandomized	University students	N=*137*	23.3	18‐49	Study-specific mind perception and support-type manipulation framework	Rule-based chatbot with Dialogflow ML^q^ intent or emotion classification	Attributing “mind” to the chatbot (explicitly or implicitly) predicted higher perceived message effectiveness (explicit *d*≈1.09; implicit *d*≈0.50); emotional support reduced message effectiveness when users did not implicitly attribute mind, whereas informational support showed no such interaction, and participants described the interaction as facilitating introspection and self-evaluation.
Lee et al, 2025 [84]	*JMIR mHealth and uHealth*	South Korea	Mixed methods	University students	N=170 (n=85; n=47; n=38)	20.6; 20.2	–	CBT-based skills and behavioral support chatbot	Semigenerative chatbot combining KoGPT2 generation, KoBERT classification, CNN^r^, and fixed CBT scenarios	Only the chatbot group showed significant improvements in time management skills, perceived stress (eg, PSS reductions of approximately 2.5‐4 points), and procrastination, with particularly strong effects in a high-engagement cluster; control participants did not exhibit comparable improvements.
Li et al, 2025 [85]	*Journal of the American Psychiatric Nurses Association*	Hong Kong	Quantitative nonrandomized	University students	N*=*30	23.03	18‐25	Mindfulness-Based Stress Reduction	Scripted rule-based chatbot using tree-graph dialogue, keyword spotting, conditional logic, and database memory	A fully chatbot-delivered audio mindfulness program produced large reductions in stress (PSS from 34.11 to 24.65; *d*≈−1.95, *P*<.001) and significant gains in related well-being domains, with high adherence (~87%) and no adverse events; most participants reported improved sleep, mood, and emotional regulation.
Ly et al, 2017 [86]	*Internet Interventions*	Sweden	Mixed methods	University students	N=37 (n=14, n=14, n=9)	21.1, 25.4, 28.8	20‐49	CBT+positive psychology	Rule-based chatbot using triggers, keywords, NLU^s^, and lightweight database	After a 2-week intervention with CBT interface (SHIMS), only the adherence group (consistent use over 2 wk) showed significant interaction effects of group and time on perceived stress (*F*_1,_ _27_=4.30; *P*=.048), with a large-medium effect size (*d*=1.06).
Marwaha et al, 2025 [87]	*The Open Psychology Journal*	India	Mixed methods	University students	N=214	–	18‐23	CBT+mindfulness	ChatGPT or LLM-based digital intervention	Following chatbot use, COVID-19 stress scores significantly declined (from 16.50 to 15.80; *P*<.01), and qualitative data indicated reduced fear of being judged, less felt need for counseling or “someone to talk to,” and fewer felt stress-related mood swings, with concomitant improvements in sleep quality.
Medeiros et al, 2022 [88]	*IEEE Transactions on Human-Machine Systems*	United Kingdom	Quantitative randomized controlled trials	Mixed adult population	N=188 (n=86; n=102*)*	28.74	18‐35	Emotion Regulation Theory (Gross) (study-specific implementation)	Hybrid chatbot using rule-based stressor mapping, NLP/text mining, Watson NLU, and ML classification	Compared with a neutral chatbot, interacting with a supportive chatbot produced modest reductions in perceived stress (~0.7 points; *P*≈.01), increased positive affect, and decreased negative affect. However, arousal-related stress indices did not show robust change.
Meng and Dai, 2021 [89]	*Journal of Computer-Mediated Communication*	United States	Quantitative randomized controlled trials	University students	N=211	20.4	–	Study-specific emotional support versus self-disclosure framework	Rule-based keyword-recognition chatbot with predefined scripts	Emotional support from the chatbot did not directly reduce stress (nonsignificant main effect) but produced significant reductions in worry and an indirect effect on stress via perceived supportiveness; receiving emotional support predicted stress reduction through feeling supported, whereas a chatbot that only self-disclosed (without emotional support) yielded the poorest stress outcomes.
Nelekar et al, 2022 [90]	*British Journal of Educational Technology*	India	Quantitative randomized controlled trials	University students	N=61	20.52	–	BDI^t^ model+ explanation-based agent architecture	Explainable rule-based BDI-style embodied agent using option-based dialogue	Stress significantly decreased across explanation conditions (eg, belief group from 7.65 to 6.91, *P*≈.01), and behavioral intentions increased for multiple health behaviors depending on explanation type; participants also reported heightened awareness of their own stress through interacting with the AI explanation system.
Orji et al, 2025 [91]	*Behaviour and Information Technology*	Canada	Mixed methods	Mixed adult population	N=190 (n=95; n=72; n=23)	–	18‐45	Positive psychology, PERMA^u^, flow theory, PSD^v^ model	Hybrid app combining smile detection (computer vision), ChatGPT API^w^, and sentiment analysis	The intervention led to significant improvements in mood (BMIS^x^ pleasantness, calmness, and positivity all increased, *d* ≈ 0.6), with 80% of interviewees reporting stress relief and relaxation; both quantitative and qualitative data indicated that the app effectively helped users feel calmer.
Park et al, 2025 [92]	*International Journal of Educational Technology in Higher Education*	South Korea	Mixed methods	University students	N=78 (n=50; n=28)	22.44; 22.6; 22.28	–	Self-reflection framework (self-disclosure–based design)	GPT-4o LLM chatbot with prompt engineering and self-disclosure manipulation	The self-disclosure chatbot elicited deeper reflective processing. This was seen in longer sessions, richer narrative content, and greater engagement across all reflection substages, which supported users’ awareness of stress triggers and appraisal patterns. Although objective assessment accuracy did not differ significantly from the non-self-disclosure version, self-disclosure interactions were perceived as more emotionally resonant and conducive to stress clarification, helping users articulate stressors and evaluate coping options more effectively than the NSD^y^ chatbot.
Park et al, 2019 [93]	*Journal of Medical Internet Research*	South Korea	Qualitative	University students	N=30	–	≥18	MI^z^	Rule-based ELIZA^aa^-style chatbot with keyword-based response generation	Participants generally appreciated evocative, context-sensitive questions that facilitated self-reflection and motivation and valued the chatbot’s nonjudgmental stance and empathy toward graduate school stress; however, some responses were seen as repetitive or generic, and many participants desired more personalized and informational support.
Qi, 2025 [94]	*International Journal of Human-Computer Interaction*	China	Quantitative nonrandomized	University students	N=206 (n=86; n=120)	19.8	–	CBT+crisis intervention components	Scripted CBT chatbot with preprepared responses	The intervention significantly reduced DASS^ab^-Stress scores (~4.2-point decrease, *P*<.01) and negative affect (SCL-90)^ac^, with most students reporting improved emotional clarity, although between-group differences in stress outcomes were not statistically significant in this sample.
Silva et al, 2020 [95]	*Journal of Medical Systems*	Portugal	Quantitative descriptive	Medical school students	N=83	22.13	17‐38	Study-specific “EUSTRESS” system (physiological stress monitoring and prediction)	Sensor-based ML stress detection using HR/HRV and neural network classification	The system effectively detected examination-related stress: physiological markers (RR^ad^ interval, SDNN^ae^, RMSSD^af^) and PSS-13 scores all indicated significantly higher stress during examinations than at baseline; a neural network model achieved good discrimination (sensitivity~75%, specificity~78%) between examination and baseline states.
Sun et al, 2023 [96]	*Frontiers in Artificial Intelligence and Applications*	The Netherlands	Quantitative randomized controlled trials	Mixed adult population	N=107 (n=30; n=77)	–	≥16	MI, BCTs^ag^, GET^ah^, mindfulness	Hybrid chatbot combining scripted expert dialogue, NLU/NLG^ai^, sentiment analysis, classification, and generation	Mindfulness-oriented chatbot conversations led to perceived stress reductions (belief in reduced stress increased; eg, stress ratings ≈3.6 vs neutral 3; *P*<.001) and strengthened beliefs in physical activity engagement for most barrier types, although in some cases control participants reported higher PA^aj^ engagement beliefs than the intervention group.
Teague et al, 2025 [97]	*Behaviour & Information Technology*	Australia	Mixed methods	Mixed adult population	N=186 (n=53; n=10; n=43; n=80)	31.2; 31.23	24‐39	CBT, mindfulness, BCT	Rule-based expert-system chatbot using hierarchical decision trees and menus	The father-focused app showed good usability (SUS^ak^≈ 75‐81; MARS^al^≈3.8‐4.3) and high engagement (~8.7 interactions per day); fathers and clinicians rated it as having high perceived impact on fathers’ mental health awareness and support-seeking, with fathers reporting clearer mood awareness and benefits from mood and goal tracking.
Tong et al, 2025 [98]	*Journal of Medical Internet Research*	Hong Kong	Quantitative randomized controlled trials	Mixed adult population	N=285	26.45	18‐61	CBT, mindfulness, behavioral activation, positive psychology	Rule-based decision-tree chatbot with predefined categorical choices	The intervention produced small to moderate improvements in self-care behaviors (*d*≈0.36), mindfulness (*d*≈0.37), depressive symptoms (*d*≈−0.26), and positive emotions (*d*≈0.28); stress decreased over time in both intervention and control groups, yielding a main effect of time but no significant group× time interaction for stress.
Troitskaya and Batkhina, 2022 [99]	*Family Process*	Russia	Quantitative randomized controlled trials	Mixed adult population	N=156 (n=58; n=98)	31.0; 29.24; 31.08	18‐54	CBT, mindfulness, positive psychology	Decision-tree chatbot with branching scripts and stored user variables	Over 21 days, participants using iCognito showed significant reductions in psychological distress (DASS-stress decreased by~2.7 points, *P*<.01) and qualitative reports of better emotional regulation and communication; there were moderate effects on relationship satisfaction and conflict reduction, although some interaction effects (eg, on quarrels and emotional intelligence) were not significant.
Weng et al, 2024 [100]	*Journal of Medical Internet Research*	Singapore	Quantitative descriptive	Mixed adult population	N=786	–	≥18	CBT, behavioral activation, mindfulness, positive psychology, motivational interviewing	Hybrid platform combining chatbot, screening algorithm, and CBT-based digital tools	Usage analytics indicated high engagement with grounding, sleep, and breathing tools, and ~57% of users reported feeling better after chatbot sessions, with greater improvements linked to completing structured CBT tools; however, outcomes were based on anonymous, uncontrolled usage data, and no RCT^am^ evidence was available.
Williams et al, 2021 [101]	*Social Sciences*	New Zealand	Mixed methods	University students	N=256 (n=124; n=132)	19.9	18‐25	CBT, DBT^an^ skills, mindfulness, behavioral activation, positive psychology	Rule-based chatbot using quick reply options and branching paths	Participants showed moderate to large pre-post improvements in stress (PSS-10 reduction of ~5.7, *P*<.001) and well-being (WHO-5), alongside modest sleep improvements and high adherence (median 19.5 of 21 days completed), with effect sizes in the small-medium range (≈0.38‐0.49).
Yong, 2025 [102]	*Journal of Technology in Behavioral Science*	France	Quantitative descriptive	University students	n=10	–	17‐21	Study-specific emotional-AI sentiment-detection+ affective computing framework	Multimodal emotional-AI system combining ML, FACS^ao^ computer vision, IoT^ap^ sensors, and smart home orchestration	AI-guided sessions improved positive affect (PANAS+6.1) and reduced negative affect (~−4.3), with users reporting relaxation, clearer mood, and interest in more personalized recommendations; however, the evidence was primarily feasibility- and experience-focused, with limited formal hypothesis testing, and the broader work emphasized integrating AI-infused mental health supports into the home.
Yoon et al, 2024 [103]	*BMJ Health & Care Informatics*	Singapore	Mixed methods	Mixed adult population	N=330 (n=278; n=12; n=40)	–	18‐60	CBT, DBT, mindfulness, behavioral activation, motivational interviewing	AI-enabled platform using chatbot with algorithmic self-assessment and resource recommendation	Platform logs showed strong engagement with breathing, grounding, reframing, and sleep tools; survey and interview data indicated reduced stress, improved emotional regulation, and high perceived usefulness, with most users finding the platform helpful (≈71%) and reporting mood improvements (≈62%); anonymity, convenience, and immediate access were valued, particularly for managing work-related stress.

a AI: artificial intelligence.

b Not applicable.

c CBT: cognitive behavioral therapy.

d NLP: natural language processing.

e LLM: large language model.

f SME: small-to-medium-sized enterprise.

g SMM: Stress Mindset Measure.

h WHO: World Health Organization.

i ACT: Acceptance and Commitment Therapy.

j HRV: heart rate variability.

k HR: heart rate.

l PSS-10: Perceived Stress Scale (10 item).

m EMI: ecological momentary intervention.

n AIML: Artificial Intelligence Markup Language.

o PFA: Psychological First Aid.

p uMARS: user version of the Mobile Application Rating Scale.

q ML: machine learning.

r CNN: convolutional neural network.

s NLU: natural language understanding.

t BDI: belief-desire-intention.

u PERMA: Positive Emotion, Engagement, Relationships, Meaning, Accomplishment model.

v PSD: Persuasive Systems Design model.

w API: Application Programming Interface.

x BMIS: Brief Mood Introspection Scale.

y NSD: non-self-disclosure.

z MI: motivational interviewing.

aa ELIZA: “ELIZA-style chatbot”, an early natural language processing computer program, reacted to user input without the capacity for logic-driven decision making, made use of pre-programmed responses.

ab DASS: Depression, Anxiety, and Stress Scale.

ac SCL-90: Symptom Checklist-90.

ad RR: R-wave to R-wave interval.

ae SDNN: standard deviation of normal-to-normal intervals.

af RMSSD: root mean square of successive differences.

ag BCT: behavior change technique.

ah GET: Graded Exercise Therapy.

ai NLG: natural language generation.

aj PA: physical activity.

ak SUS: System Usability Scale.

al MARS: Mobile Application Rating Scale.

am RCT: randomized control trial.

an DBT: Dialectical Behavior Therapy.

ao FACS: Facial Action Coding System.

ap IoT: internet of things.

Several trends are evident across the included records. First, the records were predominantly published after 2020, suggesting an increase in interest in the use of AI-enabled services for self-initiated personal stress management. Second, the included records comprised studies using qualitative research methods, as well as studies incorporating experimentation and randomized controlled trials. This indicates that the field is moving beyond early feasibility work, although the evidence base remains uneven. Subsequent research can thus build on established experimental findings and protocols to assess the efficacy of AI-enabled stress management strategies. Finally, the included records span 20 countries across multiple regions, suggesting that stress management is a concern of broad international relevance and that AI-enabled stress management strategies have been examined across diverse cultural contexts.

### Quality Appraisal

The methodological quality of the 35 included studies was assessed using the Mixed Methods Appraisal Tool (MMAT) [104], which appraises qualitative, quantitative, and mixed methods studies using 5 design-specific criteria. Two independent reviewers (the first and second authors) evaluated each study. Initial interrater agreement was 83.8%, and discrepancies were resolved through discussion. The full MMAT ratings, criteria, and coding framework applied in this review are provided in Multimedia Appendix 2.

### What Emerging Roles Do AI-Enabled Tools Play in Self-Directed Stress Management?

#### Overview

From our included records, we identified the following themes: (1) psychological intervention, use of AI to administer psychological aid for individuals independent of a clinical or medical practitioner; (2) behavioral support, where AI agents support users in making intended behavioral changes to manage stress; (3) psychoeducation, where AI-enabled agents are equipped with resources to provide users access to information about their current stressors, how to manage stress and stressors, and access to multiple sources of medical intervention; (4) companionship and emotional support, where AI-enabled agents can be present for users in moments of high stress or need, and readily provide emotional support for users; and (5) stress monitoring, detection, and triage, where AI-enabled agents assist users in monitoring personal stress levels through language detection, physiological signals when paired with wearables, and provide advice for users’ next best steps. For clarity, the term AI-enabled agents is used as an umbrella term to refer to chatbots, conversational agents, and AI-driven applications.

#### Psychological Intervention

Making use of AI-enabled agents for immediate care in high stress or prolonged stressful situations without needing to wait for a clinician or medical practitioner is a prominent benefit of AI-enabled agents. Thirteen articles found that AI can be used for stress management through providing psychological intervention enabled by AI agents [70,72-74,76,79,87,88,91-94,100]. Within these studies, AI architectures included rule-based or scripted conversational systems [70,93,94]; machine learning, recommendation, or predictive NLP-based systems [72,73,76,79,88]; LLM or generative artificial intelligence (GenAI) systems [87,92]; sensor, computer vision, or multimodal hybrid systems [74,91]; and AI-chatbot plus screening or recommendation systems [100].

In general, these AI agents have been shown to be effective in delivering timely interventions. For example, Allan [70] showed that a brief conversation with an AI chatbot shifted users’ mindsets from viewing stress as harmful to viewing stress as enhancing (*d*≈2.19), and users attributed productivity gains to this mindset change. In high-stress work environments, such as hospitals, participants who engaged with AI-supported interventions reported significant reductions in burnout, job stress, and stress responses over a 4-week period [72,73]. AI-enabled interventions for high-stress work environments were particularly valuable to users, given the fast-paced nature of their jobs, which made it unlikely that they would seek clinical intervention. Stress-response reductions were the strongest in the AI-supported intervention group [72], which supports the efficacy of immediate intervention.

AI-enabled interventions can also be combined with other forms of technology, such as biofeedback through wearables, where detecting a change in user physiology and administering immediate intervention yielded greater stress reductions in perceived stress [74]. This demonstrates the flexibility in how stress management interventions can be delivered without a clinician present. Immediate use of AI-enabled agents to mitigate stress also has positive effects. Marwaha et al [87] found a significant decline in COVID-19–related student stress scores after interacting with the chatbot compared to baseline values, with several participants qualitatively reporting reduced fear of peer judgment and decreased stress-related mood swings. Moreover, the findings of Hiller et al [76] highlight that the “just-in-time” ability of AI agents to act as a considerable merit, where users interacting with the AI-enabled system, *mila,* also saw significantly improved well-being and reduced perceived stress and depressive symptoms, with qualitative reports of improved ability to identify triggers when supported by the chatbot.

Beyond the immediate deployment of intervention, prolonged support with AI-enabled agents also benefits users by improving mood and affect. Weng et al [100] showed that users who engaged with the AI support chatbot *Wysa,* hosted on the website platform mindline.sg, repeatedly reported feeling better after chatbot sessions. Similarly, users who interacted with *SmileApp* reported significant improvements in mood, experiencing stress relief and relaxation, thereby helping them feel calmer when dealing with stressors [91].

In the context of psychological intervention, a significant number of AI-enabled systems implement evidence-based strategies derived from established frameworks such as cognitive behavioral therapy [74,76,79,87,94], mindfulness [72,73,79,87,94], and Gross’s emotion regulation theory [88]. The flexibility of chatbots to be programmed with various psychological aids and frameworks to address different scenarios and stressors demonstrates a significant use case for AI-supported agents in stress management and in catering to each user’s needs. The available evidence supports AI-enabled agents as a feasible means of delivering psychological intervention content in the reviewed contexts, particularly when systems are designed around established psychological frameworks.

#### Behavioral Support

AI-enabled agents can also help users achieve intended behaviors that mitigate stress. Ten studies found uses for AI to assist in helping users achieve behavioral changes [68,75,82,84-86,98,99,102,103]. Among the behavioral support studies, AI architectures included rule-based, scripted, or decision tree chatbot systems [75,85,86,98,99]. In these studies, AI agents primarily took the form of online chatbots, being delivered via websites or mobile apps [103] and even integrated with smart home technology [102]. Other used AI architectures include hybrid GPT or semigenerative systems combined with structured intervention content or ML classifiers [82,84] and multimodal, AI-enabled, or ML-based assistive or recommendation systems [68,102,103]. AI-supported agents were often programmed to address behaviors that contribute to stress or could improve stress outcomes. For example, Lai et al [82] showed that a 6-week intervention using an AI agent based on stress appraisal and coping theory encouraged healthier sleep habits and adaptive coping strategies, resulting in significant improvements in perceived stress, anxiety, and depressive symptoms. Participants also reported that, following interaction with the chatbot, they experienced improved sleep, mood, and emotion regulation. Another targeted behavior was procrastination. Lee et al [84] reported that engagement with a cognitive behavioral thCognitive Behavioural Therapy (CBT)–based supportive chatbot (*Moa*) was associated with greater improvements in time management skills, perceived stress, and procrastination behaviors compared with a control condition.

Beyond self-improvement domains, AI agents have helped users identify their personal conflict styles and adjust their approaches to interpersonal disagreements. For instance, Troitskaya and Batkhina [99] showed that over 21 days, using an AI-supported application was associated with a considerable reduction in psychological distress for couples. Qualitative reports indicated improved emotional regulation and communication in their relationships [99]. Consistent use of AI support was also associated with significant decreases in perceived stress while yielding improvements in mindfulness and self-care behaviors [75,84,98]. Taken together, these findings suggest that AI interventions can be effective aids in helping users make lasting behavioral changes in targeted areas, supporting them in discontinuing negative habits and encouraging positive ones. Furthermore, immediate access to AI platforms promotes greater accountability and user engagement, ensuring users remain on track to achieve their intended goals.

#### Psychoeducation

AI-enabled technology has also been applied to help users learn more about stress and stress mindsets, identify stressors, and access resources when experiencing stress. Four articles found that AI-enabled agents helped users through providing education about stress mindsets and assisting users to understand indicators of stress and how to mitigate harmful effects [69,81,97,101]. AI architectures used for psychoeducational interventions included rule-based chatbot or expert system designs [97,101], LLM- or GenAI-based support [69], and mixed app ecosystem architectures involving algorithmic chatting, AI mood checking, or possible AI recommendation features [81]. The psychoeducational functions were primarily delivered through chatbots trained on established psychological frameworks, often including CBT-informed elements, which participants engaged with independently [69,81,97,101]. For instance, Alanezi [69] reported that participants used AI agents to learn about stress and strategies for managing stressors and described experiences such as feeling heard and supported during interactions with the chatbot. The same study further noted that participants valued the ease of access to mental health resources, as well as features such as CBT-informed cognitive reframing, coping guidance, and stress self-monitoring [69]. AI-based psychoeducation can also be tailored to specific contexts or populations. Teague et al [97] reported that fathers of newborn children who interacted with a chatbot (*Rover*) trained in cognitive behavioral therapy, mindfulness, and behavior change techniques described greater awareness of their mental health and increased support-seeking. The chatbot provided information tailored to the perinatal context and was reported to support fathers in identifying, understanding, and processing mood changes associated with caring for a newborn. Participants used these outputs to contextualize their stress levels in relation to demands and plan strategies with the AI to better cope with increased stress at predicted times. Commercial apps with AI support marketed for mass population use also have the potential to impart large amounts of information to the general population. Ko and Woo [81] found that among highly rated mental health apps with AI-enabled support, frequent users reported 24-hour access and multiple sources of support and information, a significant advantage over relying on a single medical provider. Users also had access to extensive mental health information through the app, which could be tailored to their specific needs.

Moreover, findings suggest that AI-enabled technologies can effectively address population-specific stress management needs by customizing information for different groups while simultaneously broadening access to mental health information and support. Williams et al [101] reported that consistent interaction with a chatbot informed by cognitive behavioral therapy, positive psychology, and mindfulness was associated with statistically significant pre-post improvements in well-being (5-item World Health Organization Well-Being Index) and perceived stress (Perceived Stress Scale-10). These changes corresponded to small-to-medium effect sizes (approximately 0.38‐0.49) and were accompanied by self-reported improvements in adherence to healthy sleep habits and sleep quality. In summary, AI-facilitated psychoeducation can empower users to independently monitor their stress levels and manage their stress responses more effectively by teaching them evidence-based strategies. By customizing educational content to the user’s context and providing on-demand support, AI tools may help increase users’ literacy about stress and coping, which is a critical component of long-term stress management.

#### Companionship and Emotional Support

Users can make use of AI-enabled agents for immediate emotional support and companionship during high stress periods. Several studies highlighted the potential for AI agents to serve as readily available sources of emotional support [78,80,83,89,90]. Within these 5 studies, AI architectures were mixed. Two centered around LLM or GenAI conversational systems [78,80], one used a rule-based chatbot augmented by ML-based Dialogflow classification [83], one used a rule-based keyword recognition chatbot with predefined scripts [89], and one used an explainable rule-based belief-desire-intention–style embodied conversational agent [90].

The immediacy of AI-based support makes these agents valuable, especially for individuals who may be unable or reluctant to seek human support through other means. For example, Indrayanti et al [78] reported that the *PsyBot* chatbot supported users experiencing negative emotions during periods of distress, with participants describing the platform as providing a supportive space for emotional expression and feeling heard. Similarly, Nelekar et al [90] reported that participants experienced significant reductions in stress after interacting with an AI chatbot designed to facilitate conversations about users’ beliefs, desires, and life goals, alongside increased awareness and understanding of their own stress through the system’s explanation-based features.

Although participants generally report benefits from AI-mediated emotional support, some frameworks do not produce a direct reduction in stress; instead, stress may decrease indirectly via reduced worry when the chatbot increases perceived support and prompts self-disclosure [89]. In Meng and Dai [89], the Chatfuel-based supportive chatbot showed no direct effect on stress but demonstrated an indirect effect via perceived support and associated worry reductions, consistent with its design to provide emotional support or elicit self-disclosure. Within this theme, interventions grounded in established frameworks, such as *PsyBot* (Psychological First Aid) and *ARU* (Belief–Desire–Intention model with an explanation-based architecture), were associated with reductions in negative affect and stress and, in some cases, increases in positive affect; such findings highlight the value of validated psychological frameworks in guiding chatbot design in this context. Overall, the immediacy and availability of AI agents provide users with rapid access to emotional support, which may contribute to improvements in well-being over time.

#### Stress Monitoring, Detection, and Triage

AI-enabled technology can also enable users to monitor personal stress levels independently, without a clinician or other medical professional, and to detect changes in these levels before users are aware of them. Three studies fall into this category [77,95,96]. Huang et al [77] created a bespoke human-robot interaction system for adults (the *RoBoHoN*) that engaged participants in conversation. Across stress monitoring and triage studies, AI architectures included physiological or sensor-based ML systems [95] and hybrid NLP, ML, or generative systems for stressor and barrier inference [77,96]. As such, the 3 studies in this category used physiological, sensor-based, or conversational data, rather than self-reported symptom scales, to support timely remote self-intervention recommendations. Users perceived this robot as an agent capable of understanding their feelings, providing valid and reliable emotional support in moments of distress, and facilitating self-disclosure to identify causes or effects of stress. Another study by Sun et al [96] tested 6 chatbots each trained with a different therapeutic framework (eg, motivational interviewing and mindfulness). Participants in the mindfulness-oriented chatbot condition showed a significant reduction in perceived stress after the intervention, demonstrating that early detection and mitigation of stressors can be achieved with AI agents [96].

In addition to conversational monitoring approaches, Silva et al [95] described the *EuStress* solution, an AI-enabled information system designed for continuous, real-time stress assessment using wearable-derived physiological signals and machine learning classification. Consistent with a physiological stress monitoring approach, the system assesses the stress levels of the students using a wearable device to collect stress-related data with minimal user interaction. The solution was intended to interpret the stress reactivity patterns of the individual and predict stress states, cumulative effects of stress, and chronic stress, positioning the intervention as a detection-and-triage tool that translates biometric data into actionable stress-state inference [95]. While the independent use of AI-enabled systems for self-monitoring and self-management of stress is limited, the efficacy of AI in stress monitoring is supported, serving as a just-in-time strategy rather than allowing a stressful situation to spiral out of users’ control.

All 3 key studies examining AI for stress monitoring, detection, and triage [77,95,96] indicate that AI shows potential for effectively monitoring stress-related indicators and supporting stress detection and triage. *RoBoHoN*, for instance, was an interactive robotic system that inferred users’ stressors from conversational cues and was rated highly for inference accuracy [77]. These findings indicate that AI technologies can be trained to detect subtle cues indicative of stress, even before users are consciously aware of their stress. Importantly, user experiences with *RoBoHoN* were positive. Participants reported that the interaction was enjoyable and that the agent appeared to understand their feelings. They also described the agent as providing valid and reliable emotional support, which facilitated emotional self-disclosure and contributed to stress relief. In a study by Sun et al [96], a multichatbot study showed that reductions in perceived stress were accompanied by strengthened beliefs in physical activity as a stress mitigation strategy. This finding suggests that AI-based interventions may also influence users’ coping beliefs and attitudes, specifically by reinforcing exercise as a coping strategy when stress is detected. Moreover, the *EuStress* system described by Silva et al [95] further demonstrated that stress-linked physiological patterns could be meaningfully distinguished in real-world contexts, such as baseline periods versus examination periods among students. ML models were able to classify stress states with promising performance using wearable sensor data. The authors noted practical constraints, including limited sample sizes and data gaps, that affected model robustness and generalizability. These observations underscore that although AI models can detect stress, they require sufficiently large and diverse datasets to ensure reliable performance across users and contexts.

## Discussion

### Overview

The sample characteristics indicate that the included records were drawn from a reasonably broad range of participant groups but were concentrated mainly in university student samples and mixed adult populations. Several studies also focused on more specific groups, including employed nurses, small-to-medium-sized enterprise owners and managers, medical students, fathers, and children with Down syndrome and their caregivers. This suggests that the evidence base spans educational, occupational, family, and caregiving contexts. Sample sizes varied widely across the included records, from small qualitative or feasibility samples to larger intervention, survey, and platform-based samples. This pattern is consistent with the methodological range of the review, which included qualitative, mixed methods, descriptive, and trial-based studies. Where age was reported, the samples were weighted mainly toward young adults, especially in student-focused studies. Student samples were generally concentrated in the late teens to 20s, whereas occupational- and community-based samples more often fell in the late 20s to 30s. Most studies included participants aged 18 years and older, although a smaller number included adolescents or child-caregiver samples. Overall, the evidence base reflects a range of applied contexts and participant groups while remaining centered largely on students, working adults, and other accessible community populations that are well suited to the use and evaluation of AI-based stress support tools.

This review synthesized 35 studies, comprising qualitative studies (n=3), randomized controlled trials (n=11), nonrandomized quantitative studies (n=6), quantitative descriptive studies (n=4), and mixed methods studies (n=11). Collectively, the included studies examined AI-enabled tools for self-directed stress management, including chatbots, mobile apps, embodied agents, and other digital platforms. Overall, these interventions show preliminary promise as scalable and accessible supports for psychological intervention, behavioral support, psychoeducation, companionship and emotional support, and stress monitoring. However, the evidence base remains early-stage and heterogeneous, so the findings should be interpreted cautiously. Quality appraisal showed that limitations tended to cluster by study design rather than by individual study. All included studies met the 2 MMAT screening criteria (ie, “Are there clear research questions?” and “Do the collected data allow addressing the research questions?”; [104]). However, methodological quality was more mixed across the design-specific criteria. Among the qualitative studies, all 3 met all 5 study type–specific MMAT criteria. Among the randomized controlled trials, most met criteria for randomization, baseline comparability, and outcome completeness, although blinding was less consistently established and participant adherence was unclear in 1 study. For the nonrandomized quantitative studies, the main limitations were inadequate control of confounding and, in some cases, limited representativeness of the target population. For the quantitative descriptive studies, statistical analysis was generally appropriate, but measurement appropriateness and nonresponse bias were less consistently addressed. For several mixed methods studies, the main weaknesses were incomplete integration of qualitative and quantitative components and limited consideration of inconsistencies between the 2 strands. Overall, the studies were sufficient to identify emerging roles of AI in self-directed stress management, but the methodological variability warrants cautious interpretation of effectiveness and generalizability going forward.

### Key Findings

#### Overview

This review synthesized evidence on how AI can assist in self-directed stress management, identifying 5 distinct functional roles of AI-enabled agents. AI-enabled stress management systems took multiple forms, with several studies using chatbots that delivered support based on existing psychological frameworks, including CBT-informed approaches [74,76,79,87,94] and mindfulness-based programs such as Mindfulness-Based Stress Reduction [87]. Other studies evaluated web- and app-based platforms that provided self-guided tools and conversational support [75,91,100,103]. In addition, certain systems used physiological monitoring or biofeedback to support stress regulation [74,95]. These tools are largely designed to function as on-demand support for users in need, providing alerts that inform them when they experience elevated physiological symptoms indicative of stress and offering companionship in moments of emotional distress. AI-based support can be made continuously available, and because many agents are designed around established psychological frameworks, they may support users in managing immediate stressors, although evidence of effectiveness remains mixed and study dependent. Our analysis revealed clear distinctions in how AI-integrated solutions enable self-directed stress management. Each of the 5 identified themes represents distinct mechanisms or use cases for AI agents, assistants, software, and interventions. In synthesizing the emerging field, we illustrated the broad impacts and the specific ways in which AI helps users, from tracking daily habits to coping with persistent workplace stress.

Furthermore, the diversity of AI functions observed across studies was also reflected in the technical architectures and classification frameworks used to describe them. Rule-based and decision tree systems were commonly used for structured tasks, such as CBT-style exercises, mindfulness sessions, psychoeducation, and behavioral prompts. LLM- or GenAI- and NLP-enabled systems appeared more relevant where the intended role involved open-ended conversation, emotional support, or companionship. Sensor-based, physiological, computer-vision, and multimodal ML systems were more prominent when AI was used to infer stress states, emotional cues, or behavioral patterns. Many of the discussed interventions systems also integrated multiple AI approaches, making broad labels such as “chatbot” or “AI app” insufficiently precise. Although existing taxonomies classify AI systems by service design characteristics, human-AI activities [eg, 105,106], or broader risk and evaluation considerations, these frameworks remain wider in scope than this review [eg, 107,108]. By organizing studies according to AI’s functional role in self-directed stress management, this review provides a clearer and more use case–specific basis for interpreting outcomes, clarifying what each system is intended to do and avoiding the treatment of “AI” as a single undifferentiated category. In particular, a psychoeducational system, for example, may be assessed by whether it delivers accurate and understandable information; a behavioral support tool by adherence and behavior change; a psychological intervention by stress-related outcomes; a companionship and emotional support system by perceived support and emotional relief; and a monitoring or triage system by detection accuracy, timeliness, and appropriate feedback [109,110]. Across the included studies, AI served distinct functions in self-directed stress management, delivering psychological interventions, supporting behavior change, providing psychoeducation, offering emotional companionship, and monitoring or triaging stress-related states, rather than functioning as a single intervention type. This role-based structure clarifies each system’s intended purpose and offers a more meaningful basis for evaluating efficacy than AI architecture alone.

Traditionally, stress has been viewed as a negative subject, with extreme stress labeled as the cause of physical ailments and declining mental health [71,95,111]. Our findings, however, suggest that AI-based strategies aid in reframing stress in more constructive ways. For example, the use of AI tools can help individuals to reframe stress as a motivating factor [70] or as a warning sign of burnout [72], with readily available access to AI-enabled agents reducing another layer of stress in seeking clinical intervention or professional help [72]. AI-enabled stress management strategies also serve as a gateway to increasing mental health literacy about personal stressors [97], allowing users to understand their experiences from a different perspective. The functional roles identified in our review provide a clear foundation for further research on AI and stress management and will help develop more targeted solutions for each individual. Furthermore, the impact of stress-related illnesses extends beyond immediate negative affect. Stress, when unmanaged on a widespread scale, can snowball into enormous health care costs, loss in productivity at work, and, in severe cases, amount to physical disabilities [7]. The ability of AI-enabled technology to intervene and provide assistance to individuals before their stress levels reach levels of psychological or physical harm is a significant use case for AI and could be used meaningfully to help individuals manage their stress in healthy ways, while remaining accessible at any time.

#### Research Gaps and Limitations

Through this systematic synthesis, we identified several gaps in the existing literature on AI-supported self-directed stress management. First, the evidence base is dominated by early-stage pilot and feasibility studies. Although many of these studies report short-term improvements in stress-related outcomes, few evaluate mature or clinically validated systems, and distinctions between AI-enabled interventions and broader digital interventions are often insufficiently specified. As a result, evidence regarding long-term effectiveness remains limited, and this literature does not yet support meaningful estimation of pooled effect sizes. In addition, the available evidence may be skewed toward positive findings, reflecting both the novelty of AI-based interventions and broader publication biases. Second, we observed substantial heterogeneity across AI models, psychological frameworks, stress outcome measures, and evaluation methods. While this diversity reflects the field’s interdisciplinary nature, it limits comparability and makes it difficult to determine which approaches are most effective. This challenge is further compounded by the absence of standardized terminology across studies, as rapidly evolving AI capabilities and inconsistent system labeling make it difficult to systematically capture, compare, and track meaningful distinctions between intervention types over time. Third, we identified that the existing study samples are narrow and demographically skewed, with most studies involving young adults or university students. This pattern is expected given typical technology adoption trends [112], but it limits generalizability to older or less technologically proficient populations. Factors such as digital literacy and frequency of use may have differing effects on populations that were not captured within this study. Next, issues of safety, fairness, and reliability have been underexamined. Few studies systematically examined erroneous or inappropriate AI outputs, algorithmic bias, or differential performance across demographic groups. Reporting practices also tended to emphasize favorable outcomes, with comparatively little discussion of system failures, unintended effects, or potential harms. Addressing these gaps will be critical as AI-based stress management tools move toward broader real-world deployment. Finally, our search was limited to English-language publications, which introduce the potential for language bias. Both authors’ first language is English, which limits the representativeness of the evidence base outside of English.

#### Future Directions

To move the field of AI-based stress management beyond the scope of this thematic synthesis, future research should prioritize the development of a cumulative, methodologically rigorous evidence base. First, long-term randomized controlled trials are needed to assess whether stress reduction effects and behavioral changes are sustained beyond the short durations (often less than 3 months) reported in existing studies. Second, as the literature matures, meta-analytic work will be essential for estimating pooled effect sizes and identifying moderators such as age, cultural context, delivery modality, and underlying psychological frameworks. Achieving this will require more standardized outcome measures and longer follow-up intervals. Third, future studies should investigate user-AI interaction processes, including perceived mind attribution, trust, empathy, and fear of AI, as these dimensions appear to influence engagement and stress-related outcomes, in addition to examining the effects of such AI usage patterns on different age groups and demographics. Using validated scales of AI-specific individual differences, such as measures of trust, dependency, or perceived agency, would help clarify these mechanisms. Fourth, methodological standards must be strengthened across disciplines. As observed in this review, reporting practices were highly variable, and only a small subset of studies adhered to established AI-specific guidelines such as CONSORT-AI (Consolidated Standards of Reporting Trials - Artificial Intelligence) [113] and SPIRIT-AI (Standard Protocol Items: Recommendations for Interventional Trials - Artificial Intelligence) [114]. Future work should therefore prioritize transparency, include protocols for error detection and explainability, and adopt mechanisms for real-time error handling to support replicability and user safety. Fifth, improving the diversity of datasets is critical to mitigate algorithmic bias and ensure that AI systems perform equitably across populations, particularly those currently underrepresented. Sixth, the cultural adaptation of AI tools warrants systematic attention. This includes accounting for linguistic variation, culturally specific stressors, and local norms in the expression of stress and coping. The ability of different AI tools to be personalized and scaled to each individual’s needs is an area that could be explored in the future. Across the included studies, none tested age-related moderation effects. Digital literacy was not consistently considered, measured, or modeled as a covariate. One study considered digital literacy as familiarity with AI [68] but did not conduct a moderation analysis. Future research should examine whether age, digital literacy, and technological familiarity moderate engagement, acceptability, and effectiveness, given the current skew toward younger and technologically savvy populations. Seventh, research and health care organizations should begin evaluating AI-based stress interventions in real-world settings. In particular, studies set in workplaces, educational institutions, and community health platforms would be ideal for examining the extent to which emerging human-AI models improve engagement, accountability, and sustainability. Furthermore, multimodal AI systems that integrate conversational data with physiological and behavioral signals hold promise for improving ecological validity and enabling more dynamic, real-time stress detection and intervention. Together, these directions offer a roadmap for advancing the field toward more robust, inclusive, and context-sensitive AI applications in self-directed stress management.

Finally, regulation and legislation surrounding AI applications for stress management should be strengthened, especially concerning data security for clinically vulnerable populations. While AI may be beneficial to individual users, providers and governing bodies must be held accountable, ensuring users’ data is not compromised or shared without consent, so as not to harm users in the process. Existing AI guidance frameworks, including the Organisation for Economic Co-operation and Development AI Principles [115], the European Commission’s Ethics Guidelines for Trustworthy AI [116], and the ASEAN Guide on AI Governance and Ethics [117], highlight principles such as transparency, safety, privacy, human oversight, fairness, and accountability. However, these frameworks offer broad governance guidance rather than reporting standards specific to AI use in nonclinical psychological research. A potential alternative could be to adapt principles of ethical use of AI from existing sources before deploying AI in research and scaling the use of AI systems in consumer-facing products. At present, psychology-specific reporting guidance for AI use in nonclinical research appears limited. Frameworks such as CONSORT-AI [113] and SPIRIT-AI [114] offer relevant examples for AI-related reporting in clinical trial reports and protocols, but they are not general regulatory or accountability frameworks. Researchers could therefore draw cautiously on existing AI governance principles when designing studies or developing consumer-facing AI tools, particularly where human participants are involved. Human oversight may also be useful to help researchers monitor AI outputs and respond to unexpected issues during deployment.

### Conclusions

This systematic review consolidates current evidence on how AI supports self-directed stress management. We illustrated 5 distinct ways in which intelligent systems can assist users in managing stress, ranging from monitoring and intervention to behavior change and emotional support. Across these applications, one central insight emerges: several AI stress management interventions were informed by user-centered design principles or psychological frameworks that emphasize coping, behavior change, mindfulness, or emotional support [72,73,76,80,82,83]. As AI systems learn directly from their training data, model validation and reliability testing are as crucial as development studies on novel systems. Small, homogenous, or biased samples risk producing models that misrepresent or overlook entire segments of users [118,119]. As such, future work must give equal weight to large-scale, diverse data collection and rigorous validation studies, ensuring that AI tools trained for stress management generalize safely, equitably, and across contexts. At the same time, emerging techniques such as digital phenotyping [43,44] offer a promising path forward. By matching users to interventions that align with their behavioral and symptom profiles, such approaches directly address low engagement stemming from perceived unmet needs. Ultimately, these synthesized findings have wide-reaching implications across cultures, settings, and mental health domains. AI systems hold preliminary promise for expanding access and enhancing stress self-management. As these tools increasingly operate without clinician oversight, standards such as transparent communication of model capabilities, safeguards, and reporting requirements are critical to protect users while realizing the full potential of AI in self-directed interventions.

## Supplementary material

10.2196/90709 Multimedia Appendix 1 Title and abstract screening log and full-text screening log (record-level decisions), including inclusion or exclusion decisions and reasons for exclusion at full-text stage.

10.2196/90709 Multimedia Appendix 2 MMAT (2018) quality appraisal worksheet, documenting study-level methodological quality assessments for included studies.

10.2196/90709 Checklist 1 PRISMA checklist.

## Abbreviations

AI: artificial intelligence
CBT: cognitive behavioral therapy
CONSORT-AI: Consolidated Standards of Reporting Trials - Artificial Intelligence
GAIDeT: Generative Artificial Intelligence Delegation Taxonomy
GenAI: generative artificial intelligence
HIPAA: Health Insurance Portability and Accountability Act
LLM: large language model
MMAT: Mixed Methods Appraisal Tool
NLP: natural language processing
PRISMA: Preferred Reporting Items for Systematic Reviews and Meta-Analyses
SPIRIT-AI: Standard Protocol Items: Recommendations for Interventional Trials-Artificial Intelligence
WHO: World Health Organization
